# Iodine and Selenium Biofortification of Chervil Plants Treated with Silicon Nanoparticles

**DOI:** 10.3390/plants10112528

**Published:** 2021-11-20

**Authors:** Nadezhda Golubkina, Anastasia Moldovan, Mikhail Fedotov, Helene Kekina, Viktor Kharchenko, Gundar Folmanis, Andrey Alpatov, Gianluca Caruso

**Affiliations:** 1 Federal Scientific Vegetable Center, Moscow 143072, Russia; nastiamoldovan@mail.ru (A.M.); kharchenkoviktor777@gmail.com (V.K.); 2 A. Baikov Institute of Metallurgy and Material Science, Leninsky Pr. 49, Moscow 119334, Russia; Mikle_fed@mail.ru (M.F.); folmimet@yandex.ru (G.F.); aaalpatov@imet.ac.ru (A.A.); 3 Department of Hygiene, Medical Postgraduate Academy, Moscow 123995, Russia; lena.kekina@mail.ru; 4 Department of Agricultural Sciences, University of Naples Federico II, Portici, 80055 Naples, Italy; gcaruso@unina.it

**Keywords:** *Anthriscus cerefolium* L. Hoffm., Se-I biofortification, Si nanoparticles, antioxidants, mineral elements

## Abstract

Production of functional food with high levels of selenium (Se) and iodine (I) obtained via plant biofortification shows significant difficulties due to the complex interaction between the two elements. Taking into account the known beneficial effect of silicon (Si) on plant growth and development, single and joint foliar biofortification of chervil plants with potassium iodide (150 mg L^−1^) and sodium selenate (10 mg L^−1^) was carried out in a pot experiment with and without Si nanoparticles foliar supplementation. Compared to control plants, nano-Si (14 mg L^−1^) increased shoot biomass in all treatments: by 4.8 times with Si; by 2.8 times with I + Si; by 5.6 times with Se + Si; by 4.0 times with I + Se + Si. The correspondent increases in root biomass were 4.5, 8.7, 13.3 and 10.0 times, respectively. The growth stimulation effect of Se, I and I + Se treatments resulted in a 2.7, 3.5 and 3.6 times increase for chervil shoots and 1.6, 3.1 and 8.6 times for roots, respectively. Nano-Si improved I biofortification levels by twice, while I and Se enhanced the plant content of each other. All treatments decreased nitrate levels, compared to control, and increased the photopigment accumulation. Improvement of total antioxidant activity and phenolic content was recorded only under the joint application of Se + I + Si. Foliar nano-Si treatment affected other element content in plants: decreased Na^+^ accumulation in single and joint supplementation with Se and I, restored Fe, Mn and Cr amount compared to the decreased levels recorded in separately Se and I fortified plants and promoted Al accumulation both with or without Se and I biofortification. The results of this research suggest high prospects of foliar nano-Si supply for enhancing both growth and joint I/Se biofortification of chervil.

## 1. Introduction

Among the micro-elements which are essential for human beings and beneficial for plants, selenium (Se) and iodine (I) have special importance, mainly for their participation in the growth and development of all living beings, antioxidant properties, immunity optimization and protection against biotic and abiotic stresses [1]. Iodine (I) and Se deficiency are common in many worldwide areas, increasing the risk of viral (including COVID-19), oncological and cardiovascular diseases, negatively affecting fertility and restricting cognitive abilities [2,3,4]. Both Se and I are components of 3-iodothyronine deiodinases, thus taking an active part in thyroid hormones production and functioning [2,5,6]. The adequate consumption levels of Se and I are 70 µg and 150 µg per day respectively [4,7].

The importance of Se and I for human health and the close relationship of their metabolism stimulate the development of agrochemical joint biofortification of plants with these elements [1]. The micro-elements Se and I demonstrate growth stimulating properties in plants, but the range of their beneficial effect is rather narrow and specific for each species, which causes serious problems in the industrial application of the biofortification method [1]. However, low doses of Se fertilizers in a national Finnish experiment [8] revealed high possibilities of such an approach in optimizing human Se status.

To improve the efficiency of joint Se-I biofortification, phytohormones (salicylic acid [9]) and arbuscular mycorrhizal fungi [10] were applied, while vanadium (V) was proposed for increasing I accumulation in plants [1,11]. Such approaches provided moderate improvement of plant growth and a relatively moderate increase in I levels due to the sensitivity of most plants to the toxic effect of a high I dose.

In recent years, the interest in silicon (Si) application to agricultural crop productions has been increasing. Similarly to Se and I, both bulk and nano-Si are able to improve plant resistance to different forms of oxidant stress, stimulate growth, improve soil nutrient availability, plant antioxidant status [12,13,14], and participate in the transpiration process [15]. The small size of Si nanoparticles and immense surface area reportedly provide higher potential in agricultural application than bulk silicon for improved stress tolerance, nutrient accumulation, and plant growth [13]. These facts indicate possible prospects of nano-silicon application in Se and I biofortification processes. The improvement of rice growth due to foliar Se and Si nanoparticles supplementation under salinity stress [12], as well as the beneficial effect of these elements on the production of other plant species [16,17,18,19], provides the basis for investigating further opportunities in this direction. Restricted data regarding the efficiency of foliar nano-Si supplementation [19,20,21,22], scant information on the mechanism of the Se-Si relationship [23], and lack of information relevant to the I-Si interaction, especially the nano-Si effect, suggest the need to appropriately investigate the interaction peculiarities between these elements.

The present work aimed to evaluate the efficiency of chervil plant biofortification with Se and I under foliar nano-Si supply and the effects of these micro-elements on plant biochemical characteristics and elemental composition. Chervil (*Anthriscus cerefolium* (L.) Hoffm.) belongs to the Apiaceae family and is considered an exclusive spice with a short vegetation period, and remarkable medicinal properties and antioxidant content [24].

## 2. Results and Discussion

To evaluate the relationship between Se, I and nano-Si in chervil, nano-Si, sodium selenate and potassium iodide solutions were supplied through foliar applications, taking into account that this approach allows for minimizing the effect of soil characteristics on the interactions between the aforementioned elements. Besides, foliar biofortification is reportedly beneficial for supplying I to leafy crops [25] and is widely used for Se biofortification [26]. Among Se chemical forms used for plant biofortification, sodium selenate (Se^+6^) was chosen because more mobile and less toxic compared to selenite form (Se^+4^). Previous reports related to the high efficiency of nano-Si foliar application [21] set up the basis of nano-Si application in Se/I biofortification of chervil.

### 2.1. Biometrical Parameters, Dry Matter and Nitrates

Separate and joint foliar application of Se and I at concentrations of 10 mg L^−1^ and 150 mg L^−1^, respectively, were beneficial for chervil growth, with remarkable increases in shoot and root biomass (Table 1). The data reported in Table 1 indicate higher shoot/root biomass under I supplementation compared to Se application (3.4–3.5 and 2.7 times increase in shoots and 3.1 and 1.6 times increase in root biomass, respectively). Interestingly, higher concentrations of sodium selenate applied to plants in our previous research (25, 50, 75 mg L^−1^ compared to 10 mg L^−1^ in the present work) did not improve chervil growth [27], thus suggesting the higher effectiveness of low Se doses to *Anthriscus cerefolium* L.

**Figure 1:**
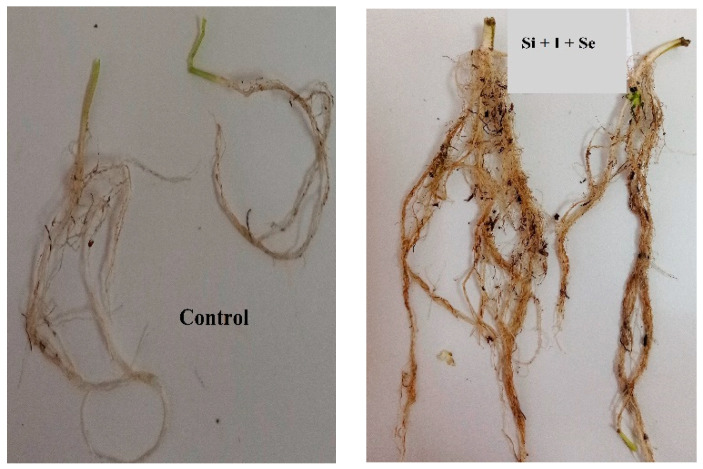
Root system of chervil under I, Se and Si treatments as compared to control.

In the present conditions of Se and I biofortification, foliar application of nano-Si gave additional benefits causing shoot and especially root growth stimulation (Figure 1), proving 8.2 and 3.8 times increase in root biomass of Se and I treated plants, respectively, and 2.0 and 1.8 times increase in shoot biomass, compared to plants treated separately with Se and I.

Indeed, the mean increase in root length by 1.4 times was recorded upon foliar nano-Si application, while the shoot length in the same conditions increased only by 1.16 times. Nano-Si foliar application elicited a biomass increase in chervil by 4.8 times for shoots and 5.7 times for roots. The most pronounced increase in root biomass was recorded upon Si application in combination with Se and/or I, and reached 12 times increase under joint application of Si and I.

Neither Se, nor I or Si are considered essential for plants, but are able to stimulate their growth only in special conditions [28]. Chervil belongs to a group of Se non-accumulators-plants, highly sensitive to Se toxicity, and therefore the growth stimulation effect is achieved mainly at low Se concentrations, capable to optimize water and nutrient supply and reduce oxidant stress [28].

A recent investigation of iodine role in plants revealed that the beneficial effect of I supplementation may relate to the incorporation of I into plant proteins, thus affecting plant growth and development [29]. The latter outcome is in agreement with the revealed Se and I growth stimulation effect in chervil.

According to the literature reports, the beneficial effect of Si is recorded mainly under stress conditions, with great differences among different species, while in normal conditions Si supplementation has little or no effect [30]. Furthermore, previous findings indicate that Si may improve plant nutrition in non-stressed conditions [31]. Notably, the mentioned conclusions relate mainly to soil bulk Si application, while many fewer investigations were devoted to foliar nano-Si supply [22].

An increase in root and shoot biomass in conditions of Se and/or I biofortification, with or without nano-Si supply, indicates a lack of significant oxidant stress during plant growth and development, excluding the stress possibility connected with Se, I or nano-Si application. The lack of significant changes in plant dry matter content in most treatments is consistent with the latter statement. On the other hand, we do not have enough information about interspecies differences in Se, I and nano-Si interaction under foliar application of these elements. Previous outcome regarding soil bulk Si supplementation suggests great differences in various species resistance to Si [28,31]. Furthermore, the low concentration of nano-Si in the present study (14 mg L^−1^), compared to those described in the literature [12,21,32], entails that the detected growth stimulation effect is not directly connected with Si level increase in plant tissues but is of minor importance. To reveal the mechanism of the Si effect, additional comparative investigations are necessary for soil and foliar Se/I biofortification of different species under the application of both nano- and bulk Si.

**Figure 2:**
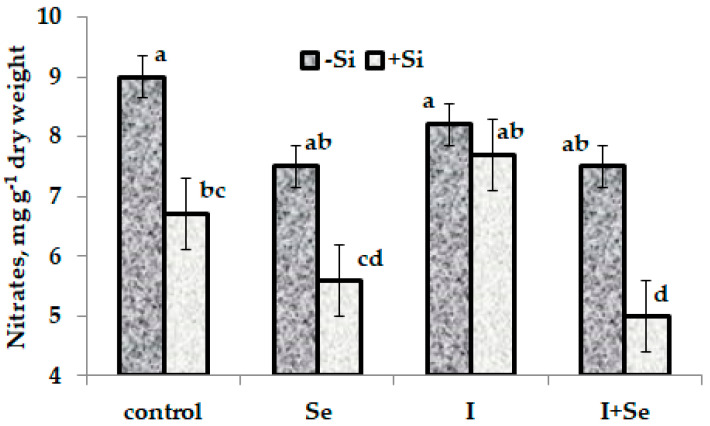
Effect of Si on nitrate accumulation in control chervil plants and plants fortified with I and/or Se. Values with the same letter do not differ statistically according to Duncan test at *p* < 0.05.

Participation of Se, I and Si in nitrogen metabolism was recorded through nitrate accumulation levels (Figure 2). Lack of any nitrate level change in plants treated with I and a tendency of nitrate concentration to decrease under Se biofortification are in agreement both with previous observations of I biofortification of radish [33] and with studies of Se effect on nitrate reductase activity [34].

Though both nano- and bulk Si are known to improve nitrogen uptake, assimilation, remobilization and induce amino acids biosynthesis under soil application [35,36,37,38], a significant decrease in nitrate accumulation in chervil treated with nano-Si, Se + nano-Si and Se + I + nano-Si is the first observation of foliar nano-Si effect on nitrate metabolism. Lack of any effect of nano-Si on nitrate accumulation in chervil under I supply demonstrates a substantial different interaction between Se and nano-Si, and between I and nano-Si. In this respect, further investigations including a comparison between nano- and bulk Si treatments are needed to unveil peculiarities of Se-I-Si interaction.

### 2.2. Photosynthetic Pigments

In the present experiment, single and joint biofortification of chervil with I and Se were characterized only by a tendency to the improvement of photosynthetic pigment synthesis. The non-significant effect of Se on photopigment biosynthesis may relate to the low applied concentration of this element. Indeed, the statistically significant beneficial effect of Se on chlorophyll and carotene accumulation in chervil leaves was previously recorded at concentrations equal to or higher than 50 mg Se L^−1^ [27], proving the participation of Se in chlorophyll biosynthesis [39]. The effect of I on photosynthesis is not predictable, as previously reported [40,41].

According to the literature, both soil and foliar application of nano-Si enhances the accumulation of photosynthetic pigments either under stress [13] or normal conditions of vegetation [21]. The present results are consistent with the latter observations indicating the predominant role of nano-Si treatment on photopigments’ accumulation (Table 2). Indeed, the mean increase in total chlorophyll content in plants subjected to foliar nano-Si exceeds by 18% that recorded in chervil without application of Si.

### 2.3. Total Antioxidant Activity and Phenolic Content

Se, I, and bulk and nano-Si are able to increase the plant antioxidant status, providing resistance to stress factors [1,13]. The lack of significant stress factors in the present experiment may be the reason of the low changes in the antioxidant status of chervil subjected to different treatments (Table 3). Total antioxidant activity (AOA) increased significantly only in the case of joint Se + I + Si application, whereas ascorbic acid content reached the highest value under joint I + Si supplementation. The lack of correlation between ascorbic acid content and total antioxidant activity may be explained by the fact that ascorbic acid represents water-soluble antioxidants, while AOA of ethanolic extracts of plants measured in the present investigation reflects the lipid-soluble fraction. It seems obvious that, compared to normal vegetation conditions, stress factors may strengthen the relationship of Se, I and nano-Si, which would guarantee a more intensive beneficial effect of these elements.

### 2.4. Mineral Composition

The mineral composition of plants biofortified with various elements provides a valuable evaluation of the quality characteristics of functional food produced. Despite the importance of this topic, the relationships between Se, I, macro- and micro-elements, as well as toxic elements in plants, have been scantly investigated. In the latter respect, the accumulation of 25 elements in chervil shoots was analyzed and, among the detected differences in macro- and micro-element content, the most important ones related to Se and I.

#### 2.4.1. Se, I and Si

The data presented in Table 4 indicate beneficial interactions between Se and I, considering that supplying each of the two elements increased the accumulation of the other. Contrary, no synergistic effect between Se and I was recorded under joint biofortification of chervil with I and Se. Though Se-I interaction in plants remains poorly investigated, the synergistic effect between Se and I was demonstrated under the joint Se + I biofortification of chickpea [10] and Indian mustard [42]. Nevertheless, other agricultural crops did not reveal such a relationship [1].

**Figure 3:**
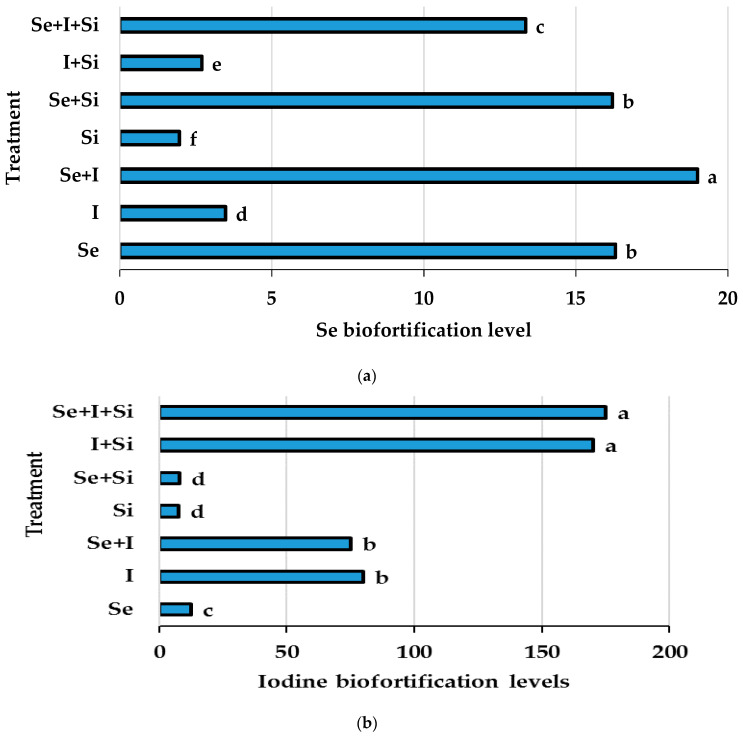
Biofortification levels (BL) of Se (**a**) and I (**b**) in chervil shoots under single and joint application of Se, I and Si (BF is equal to the ratio between element content in the treated and control plants). Values with the same letter do not differ statistically according to Duncan test at *p* < 0.05.

In the present investigation, the low concentrations of Si did not elicit significant changes in Si levels in chervil shoots (Table 4). However, nano-Si foliar supply was highly valuable for modulating I but not Se accumulation (Table 4, Figure 3). Indeed, the application of nano-Si increased I accumulation in chervil by 7.5 times, while joint applications of I and I + Se with Si increased I concentration by almost twice compared to a single I application (Figure 3). These results are especially important, as the detected phenomenon is accompanied by plant growth stimulation, contrary to previous works devoted to joint Se + I biofortification, where the increase in I concentration was limited by I toxicity [1]. The comparison of the present results with previous attempts to improve I accumulation upon joint Se + I supplementation, via utilization of salicylic acid [11], vanadium application [9], soil administration of marine algae [41,42] and AMF inoculation [10], suggests high prospects of foliar nano-Si supplementation for production of chervil with elevated levels of Se and I.

As shown in Figure 3a,b, the highest value of Se biofortification was recorded with Se + I application, while the highest I biofortification levels were recorded under foliar nano-Si supply.

Among Se, I and Si relationships in chervil, a small but statistically significant decrease in Si concentration in plants subjected to joint Se + I biofortification was recorded (Table 4); the latter is predominantly connected with the joint application of Se and I, as nano-Si supply did not affect this element concentration due to the low dose. Previous investigations of Indian mustard [42] and chickpea [10] biofortification with I and Se did not reveal any effect of Se and I on Si accumulation [42], which makes it suppose the importance of genetic factors in Se, I and nano-Si interaction. Indeed, these plants differ significantly in the ability to accumulate Se and other macro- and micro-elements, in protein content, resistance to oxidant stress, and have different edible plant parts, such as leaves in chervil and Indian mustard, seeds in chickpea.

Taking into account the non-significant Si accumulation in chervil plants as a result of low Si dose applied, its beneficial effect on growth, iodine accumulation and nutritional value of this crop is surprising. Indeed, most of investigations which reported Si growth promoting activity focused on the application of high Si concentrations, resulting in significant plant accumulation of this element [43]. In this respect, the close relationship between Si and plant hormonal status may be a possible explanation of this phenomenon. Indeed, silicon supplementation is known to cause significant changes in jasmonic and salicylic acid, auxin and cytokinin biosynthesis [39]. Furthermore, foliar application of low doses of nano-SiO_2_ on cucumber was shown to greatly affect phytohormones’ content: increase in jasmonic and abscisic acid content and decrease in indolyl acetic acid [19]. Nano-SiO_2_ significantly decreased Cd accumulation in cucumber roots, despite the lack of changes in plant Si levels [19]. Soil application of nano-SiO_2_ to cotton at 10 mg L^−1^ concentration did not cause changes in plant Si levels but significantly increased indolyl acetic acid production [20]. In the present experiment, nano-Si elicited growth-promoting effect and biochemical changes in chervil also under low Si dose. Despite the scant data relevant to phytohormone effect on I accumulation [9,11,29], it may be supposed that, at least partially, the Si-I interaction can be achieved through plant hormonal status modification, though additional investigations are needed to confirm this hypothesis.

Considering the Se and I levels in Se/I fortified chervil shoots, the latter may be deemed a new functional food with a significant content of Se and I. Taking into account the adequate Se and I consumption levels (ACL), 50 g of fresh biofortified chervil shoots can provide up to 25% of Se ACL (37 µg) and 100% of I ACL, which widens the list of Se/I biofortified vegetables produced up-to-date [1,9,11].

#### 2.4.2. Macro-Elements

Different investigations indicate that Se and I biofortification, as well as bulk and nano-Si application, may affect macro- and trace elements accumulation in plants [10,23,40]. The data reported in Table 5 reveal that among macro-elements significant changes occurred predominantly for Na, whereas the concentrations of essential elements, such as P, K, Ca and Mg, were not generally affected by Se, I or Si content in chervil shoots.

Previous investigations of I biofortification peculiarities revealed that potassium iodide application reduced Na accumulation in lettuce seedlings [44]; the latter outcome is consistent with the Na content decrease by almost twice recorded in chervil treated with I, in the present research. Literature reports indicate that both Se and I can alleviate salt stress in plants [45,46].

A high K^+^/Na^+^ ratio in the cytosol guarantees plant resistance to salt stress [47]. The data reported in Table 5 indicate that the highest K/Na ratios relate to the joint application of Si with Se and/or I (142–200 compared to 83 in control plants), whereas a much smaller effect was recorded under Se, Se + I or Si supplementation (K/Na ratio equal to 116). Indeed, Na^+^ content decreased by 2.66 times in the case of Se + Si application, by 1.6 times under I + Si and Si supplementation and by 1.9 times upon joint Se + I + Si application. This phenomenon demonstrates additional benefits in Se and I joint utilization under foliar nano-Si supply. Up to date, alleviation of plant salt stress using nano-Si was reported only in the case of soil Si supplementation, which reduced NaCl accumulation, thus promoting root growth [48,49] and decreasing root-to-shoot translocation of Na^+^ [50]. Notably, studies of Si interaction with other macro-elements were carried out only on soil application of bulk Si [23], while no information is available on the foliar effect of nano-Si.

#### 2.4.3. Micro-Elements

To date, there are extremely scant data regarding the interaction of I with other elements [51], while Se supplementation effect on plant mineral composition greatly varied depending on the species investigated, methods and doses of Se supplementation [52]. The biofortification of chervil with I and Se resulted in a decrease in Fe, Mn, Zn, Mo and Co, while joint application of Se and I did not change the concentration of these elements. The ability of I to reduce the concentration of Fe, Mn and Zn was previously demonstrated in lettuce seedlings [44]. Controversial data were obtained with other agricultural crops: Se/I biofortification of chickpea resulted in no changes of the element content [10]; no effect on micro-elements content was recorded also in shallot [53], onion and garlic [54], fortified with Se, while an increase in the concentration of Fe, Mn and Zn was recorded in Indian mustard under foliar Se and I biofortification [42].

**Figure 4:**
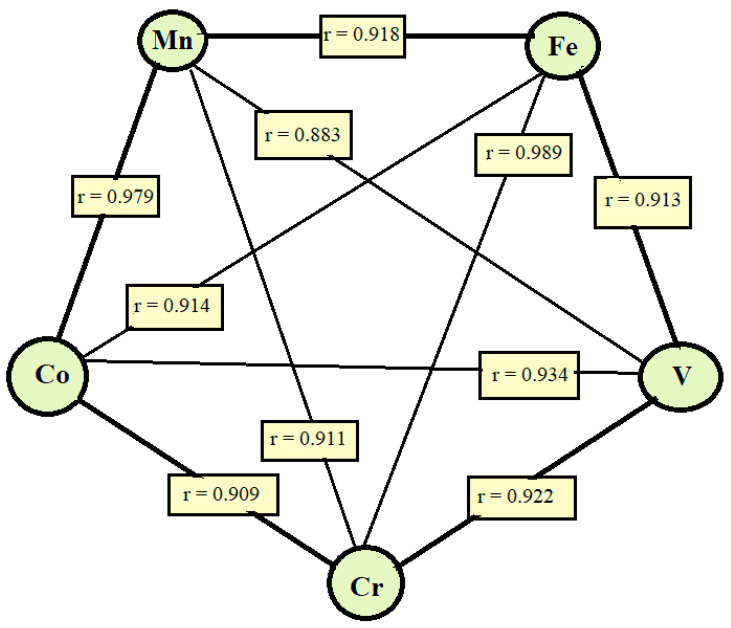
The most intensive relationships between the elements investigated in chervil shoots (*p* < 0.001).

The present results on chervil biofortification with Se and I indicated for the first time the importance of foliar nano-Si application in restoring Fe and Mn levels in plants (Table 6). The known relationship between Fe and Mn was confirmed by the Mn level changes, where the highest content was 93.7 mg kg^−1^ d.w. in the case of nano-Si application, compared to 1.5-times lower values in control plants. The coefficient of correlation between Fe and Mn was equal to 0.918 (*p* < 0.01) (Figure 4). Up-to-date only one work devoted to foliar application of nano-Si on rice seedlings reported changes in micro-element content as affected by nano-Si and Cd application [55], where an increase in Mg, Fe and Zn content was detected [55].

#### 2.4.4. As, Al, Cd, Cr, Ni, Pb, Sr and V

It is well known that Se is an important antagonist of toxic metals and metalloids [52,56]. Indeed, Se biofortification resulted in a significant decrease in As, Cr, Pb, Sr and V content in chervil shoots, even without toxic element uptake (Table 7). Up to date, there are extremely scant data relevant to the interaction of I with other elements [51]. In the present study, I treatment resulted in a decrease in Cr and Pb in chervil shoots, while no changes were observed in plants subjected to joint supplementation with I and Se (Table 7).

In agriculture, Si nanoparticles are highly valuable in plant protection against metal toxicity [57], though the protection effect closely relates to soil application. The foliar nano-Si application showed the most interesting beneficial effects on V levels (Table 7), as the latter is known to participate in I, Cr and Al accumulation. Nano-Si application to chervil resulted in restoration of Cr content in plants, which decreased due to Se and I biofortification, a significant increase in I accumulation (Table 4) and pronounced Al content rise (Table 7). The phenomenon of V, Cr and Al increase does not cause any ecological risks due to rather low levels of these elements and indicates the possibility of nano-Si participation in the element distribution. Notably, foliar nano-Si effect on Al accumulation in chervil plants contrasts with the Al accumulation decrease under soil Si application [43].

#### 2.4.5. Correlations

The coefficients of correlations between the analyzed mineral elements in chervil shoots provided a complex frame of interactions (Supplement Table S1), the most intensive ones being between Mn, Fe, V, Cr and Co (Figure 4), while no significant correlations were detected for B and Mg. The lack of correlations between the mentioned elements and the foliar-supplied ones (Se, I, Si) suggests the indirect effect of Se, I and Si, on plant elemental status.

## 3. Materials and Methods

### 3.1. Growing Conditions and Experimental Protocol

The experiment was conducted in 2020 and 2021 at the Federal Scientific Vegetable Center (Moscow region; 55°39′23″ N, 37°12′43″ E), in plastic pots using chervil seeds of the genotype 21–20 sown on 6 June. The values of mean temperature and relative humidity, and total photosynthetically active radiation (PAR) during the vegetation period were: in 2020, 21.0 °C, 73.0% and 293 MJ m^−2^ in June, and 23.8 °C, 74.9% and 244 MJ m^−2^ in July; in 2021, 17.2 °C, 69% and 285 MJ m^−2^ in June, and 20.1 °C, 72.0% and 232 MJ m^−2^ in July. The photoperiod duration (day length) was 17.20–17.33 in June and 17.26–16.0 in July.

Each pot (10 L volume, 25 cm diameter, 20 cm height) contained a field clay-loam soil, with the following characteristics: pH 6.8, 2.1% organic matter; 1.32 mg-eq 100 g^−1^ hydrolytic acidity; 18.5 mg kg^−1^ mineral nitrogen; 21.3 mg kg^−1^ ammonium nitrogen; 402 mg kg^−1^ mobile phosphorous; 198 mg kg^−1^ exchangeable potassium; sum of absorbed bases as much as 93.6%; cation exchange capacity as much as 15 mg-eq 100 g^−1^ soil; soil moisture field capacity as much as 25.2%.

All the pots were placed outdoors under a plastic shelter protecting from rainwater. Each pot had a tray for collecting leaching water, which was recycled during the next irrigation. The experimental protocol included eight treatments: (1) control (without any supplementation); (2) foliar supplementation of Na_2_SeO_4_ solution (10 µg L^−1^); (3) foliar supplementation of KI (150 mg L^−1^); (4) foliar supplementation of nano-Si (14 mg L^−1^); (5) foliar supplementation of Na_2_SeO_4_ and KI with the above-indicated concentrations; (6) foliar supplementation of Na_2_SeO_4_ + nano-Si with the above-indicated concentrations; (7) supplementation of KI + nano-Si with the above-indicated concentrations; and (8) foliar supplementation of Na_2_SeO_4_ + KI + nano-Si with the above-indicated concentrations. The volume of solutions per pot was 50 mL. A randomized complete block design was used for the treatment distribution in the field, with three replicates, and each treatment consisted of four pots. On 6 June, 30 chervil seeds were sown in each pot, with a density of 16 pots per m^2^. The plants were watered three times a week to prevent any water stress during the whole growing period. On 5 August, all plants were harvested, separated into shoots and roots, washed with tap water, rinsed several times with distilled water, dried with filter paper and weighed to assess the biomass, dried up to constant weight at 70 °C and homogenized.

### 3.2. Colloidal Solution of Silicon Nanoparticles

#### 3.2.1. Laser Ablation in Liquid (LAL)

Si nanoparticles in the form of a colloidal solution were obtained by pulsed laser ablation in a liquid (LAL). The irradiation was achieved by using a pulsed nanosecond Nd:YAG laser with a wavelength λ = 1064 nm. The laser pulse length was 12 ns and pulses frequency—1 Hz. Rated energy in the pulse was 2.5 J. As a target, special-purity grade single-crystalline silicon was sprayed. The target was immersed in a static glass cell with 250 mL of deionized water. The laser beam was focused on target inside the cell by lens. The target was irradiated for 30 min without stirring.

#### 3.2.2. Inductively Coupled Plasma Atomic Emission Spectrometry (ICP-AES)

The silicon concentrations in the obtained colloidal solutions were measured by inductively coupled plasma atomic emission spectrometry (ICP-AES) with an ULTIMA 2 (Horiba Jobin Yvon, France) spectrometer. Monochromator—holographic grating 2400 lines mm^−1^, focal length 1 m. Sample preparation consisted of sequential dilution of the initial colloidal solutions with high purity grade water by a factor of 5 or more until the required concentration was obtained. Diluted solutions were sprayed into the plasma torch of the spectrometer by Meinhard atomizer. Colloidal solutions of silicon in water do not require preliminary dissolution of samples. For the determination of silicon, the most sensitive analytical line at 251.611 nm was chosen. The aqueous base of solutions, as well as the presence of one element in the solution, makes it possible to determine the concentration of silicon without the influence of other elements.

#### 3.2.3. Dynamic Light Scattering (DLS) and Z-Potential

Sizes of the dispersed phase particles were measured by dynamic light scattering (DLS) method using Photocor Compact Z (Photocor, USA) laser analyzer with a wavelength λ = 589 nm and laser rated-power output of 32 mW. To estimate the surface charge of particles of colloidal solution, the magnitude of the electrokinetic potential (ζ, mV) was determined via electrophoresis using the same instrument. To this end, 2 mL of colloidal solution was added in a quartz cell at 25 °C for the analysis.

**Figure 5:**
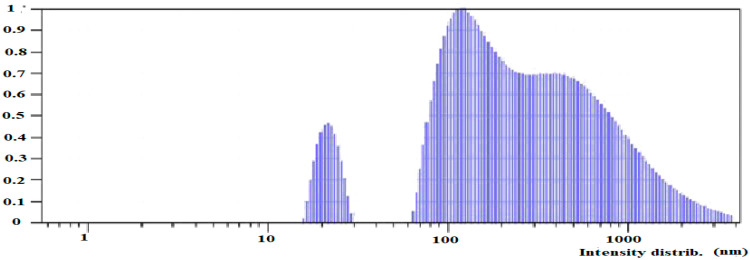
Silicon particle size distribution of freshly prepared colloidal solution.

The concentration of silicon in the colloidal solution was equal to 14 mg L^−1^. To assess the aging of the colloidal solution, two types of solutions were analyzed. Figure 5 shows the particle size distribution in a freshly prepared colloidal solution.

**Figure 6:**
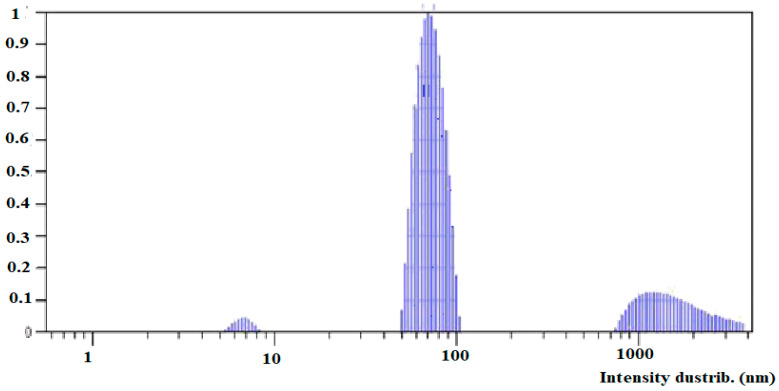
Silicon particle size distribution of aged colloidal solution.

The sedimentation process in the freshly prepared solution results in a wide particle size distribution. The first relatively narrow peak shows mean particle size of about 22 nm. The second range is from 80 nm to 3 μm. The second type of solution was settled for 1 month (Figure 6).

Figure 6 shows 3 peaks with mean particle size of 7 nm, 72 nm and 1.6 μm. The last peak was formed after vigorous stirring of the solution. The ζ-potential of the aged and new solution did not differ significantly and was estimated at about −33.5 mV.

### 3.3. Biochemical Analyses and Elemental Composition of Plants

#### 3.3.1. Dry Matter

The dry matter was assessed gravimetrically by drying the samples in an oven at 70 °C until constant weight.

#### 3.3.2. Photosynthetic Pigments

Half a gram of fresh leaf sample was homogenized in a porcelain mortar with 10 mL of 96% ethanol. The homogenized sample mixture was quantitatively transferred to a volumetric flask, bringing the volume to 25 mL and the mixture was filtered through filter paper. The resulting solution was analyzed for Chlorophyll-a, Chlorophyll-b and carotene determination through a spectrophotometer (Unico 2804 UV, USA). Calculation of chlorophyll and carotene concentrations was achieved using appropriate equations [58]:

Ch-a = 13.36A_664_ − 5.19A_649_;

Ch-b = 27.43A_649_ − 8.12A_664_;

C c = (1000A_470_ − 2.13 Ch-a − 87.63 Ch-b)/209;

where A = Absorbance, Ch-a = Chlorophyll a, Ch-b = Chlorophyll b and C c = Carotene.

#### 3.3.3. Ascorbic Acid

The ascorbic acid content was determined by visual titration of plant extracts in 6% trichloracetic acid with Tillman’s reagent [59]. Three grams of fresh chervil leaves were homogenized in a porcelain mortar with 5 mL of 6% trichloracetic acid and quantitatively transferred to a measuring cylinder. The volume was brought to 60 mL using trichloracetic acid, and the mixture was filtered through filter paper 15 min later. The concentration of ascorbic acid was determined from the amount of Tillman’s reagent that went into titration of the sample.

#### 3.3.4. Preparation of Ethanolic Extracts

Half a gram of dry homogenized chervil shoots was extracted with 20 mL of 70% ethanol at 80 °C over 1 h. The mixture was cooled and quantitatively transferred to a volumetric flask, and the volume was adjusted to 25 mL. The mixture was filtered through filter paper and used further for the determination of polyphenols (TP) and total antioxidant activity (AOA) [60].

#### 3.3.5. Total Polyphenols (TP)

Polyphenols were determined spectrophotometrically based on the Folin–Ciocalteu colorimetric method according to Golubkina et al. [59].

The concentration of polyphenols was calculated according to the absorption of the reaction mixture at 730 nm using 0.02% gallic acid as an external standard. The results were expressed in mg of gallic acid equivalent per g of dry weight (mg GAE g^−1^ d.w.)

#### 3.3.6. Antioxidant Activity (AOA)

The antioxidant activity was measured via titration of 0.01 N KMnO4 solution with ethanolic extracts of dry samples [59].

#### 3.3.7. Nitrates

Nitrates were assessed using ion-selective electrodes of an ionomer Expert-001 (Econix Inc., Moscow, Russia) [27]. Five grams of fresh chervil shoots were homogenized with 50 mL of distilled water. Forty-five mL of the resulting extract were mixed with 5 mL of 0.5 M potassium sulfate background solution (necessary for regulating the ionic strength) and analyzed through an ionomer for nitrate determination.

#### 3.3.8. Element Composition

Al, As, B, Ca, Cd, Co, Cr, Cu, Fe, Hg, K, Li, Mg, Mn, Na, Ni, P, Pb, Si, Sn, Sr, V, and Zn contents in dried homogenized samples were assessed using ICP-MS on quadruple mass-spectrometer Nexion 300D (Perkin Elmer Inc., Shelton, CT, USA), equipped with the seven-port FAST valve and ESI SC DX4 autosampler (Elemental Scientific Inc., Omaha, NE, USA) at the Biotic Medicine Center (Moscow, Russia). Rhodium 103 Rh was used as an internal standard to eliminate instability during measurements. Quantitation was performed using external standard (Merck IV, multi-element standard solution); Perkin–Elmer standard solutions for P, Si, and V, and all the standard curves were obtained at five different concentrations. For quality control purposes, internal controls and reference materials were tested together with the samples daily. Microwave digestion of samples was carried out with sub-boiled HNO_3_ diluted 1:150 with distilled deionized water (Fluka No. 02, 650 Sigma-Aldrich, Co., Saint Louis, MO, USA) in the Berghof SW-4 DAP-40 microwave system (Berghof Products + Instruments Gmb H, 72, 800 Eningen, Germany). The instrument conditions and acquisition parameters were: plasma power and argon flow, 1500 and 18 L min^−1^, respectively; aux argon flow, 1.6 L min^−1^; nebulizer argon flow, 0.98 L min^−1^; sample introduction system, ESI ST PFA concentric nebulizer and ESI PFA cyclonic spray chamber (Elemental Scientific Inc., Omaha, NE, USA); sampler and slimmer cone material, platinum; injector, ESI Quartz 2.0 mm I.D.; sample flow, 637 L min^−1^; internal standard flow, 84 L min^−1^; dwell time and acquisition mode, 10–100 ms and peak hopping for all analytes; sweeps per reading, 1; reading per replicate, 10; replicate number, 3; DRC mode, 0.55 mL min^−1^ ammonia (294993-Aldrich Sigma-Aldrich, Co., St. Louis, MO 63103, USA) for Ca, K, Na, Fe, Cr, V, optimized individually for RPa and RPq; STD mode, for the rest of analytes at RPa = 0 and RPq = 0.25 [61].

Trace levels of Hg and Sn in samples were not taken into account and, accordingly, they were not included in the tables.

#### 3.3.9. Determination of Iodine

Determination of iodine was carried out according to [60], using the Voltamperometric Analyzer TA-4 (Tomanalyte, Tomsk, Russia) equipped with built-in UV lamp and three-electrode electrochemical cell: auxiliary and reference electrodes (silver chlorides in 1 M KCl), and working electrode, i.e., a modified silver electrode. Then, 2 mL of 10% KOH solution was added to 0.1 g of dried homogenized samples, which were ashed at 40–550 °C. The mixtures were cooled down, 1 mL of 10% zinc sulfate solution was added, and ashing was repeated in the same mode. The resulting white probes were dissolved in 10 mL of distilled water, and iodine concentration was determined using concentrated formic acid as a background electrolyte and standard potassium iodide solutions of 0.1 mg·L^−1^, 1 mg·L^−1^, and 10 mg·L^−1^.

#### 3.3.10. Determination of Selenium

Selenium was analyzed using the fluorimetric method previously described for tissues and biological fluids [62]. Dried homogenized samples were digested via heating with a mixture of nitric and chloral acids, subsequent reduction of selenate (Se^+6^) to selenite (Se^+4^) with a solution of 6 N HCl, and formation of a complex between Se^+4^ and 2,3-diaminonaphtalene. Calculation of the Se concentration was achieved by recording the piazoselenol fluorescence value in hexane at 519 nm λ emission and 376 nm λ excitation. Each determination was performed in triplicate. The precision of the results was verified using a reference standard of Se fortified chervil stem powder in each determination with a Se concentration of 1865 μg Kg^−1^ (Federal Scientific Vegetable Center).

#### 3.3.11. Biofortification Values

The evaluation of the efficiency of Se and I biofortification of chervil shoots was achieved using the so-called “biofortification level” (BL), which was calculated according to the equation:

BL = C_1_/C_2_,

where C_1_ is the concentration of Se/I in leaves of biofortified plants and C_2_ the concentration of Se/I in leaves of control plants.

### 3.4. Statistical Analysis

Data were processed by analysis of variance, and mean separations were performed through Duncan’s multiple range test, with reference to the 0.05 probability level, using SPSS software version 21. Data expressed as percentages were subjected to angular transformation before processing.

## 4. Conclusions

The results of the present investigation provide the first information regarding chervil Se and I biofortification efficiency with and without foliar nano-Si supply, emphasizing the high benefits of foliar nano-Si application for increasing I concentration and improving plant yield. Foliar nano-Si supplementation to chervil plants produced the following effects, compared to the single Se and I treatments: (i) Enhanced photosynthetic pigment production; (ii) Decreased nitrate and Na^+^ contents; (iii) Restored Fe, Mn and Cr accumulation. Al content was also increased by foliar nano-Si application under Se + I biofortification. Considering the high variability of the Si effect on different plant species, further studies will be planned to unveil the general prospects of both soil nano-Si application for strengthening plant Se/I biofortification, and foliar nano-Si effect on Se/I biofortification of other agricultural crops.

## Figures and Tables

**Table 1:** Yield, dry matter, and biometrical parameters of chervil under Se, I and Si supplementation.

Treatment	Length (cm)		Yield (g·m^−2^)		Dry Matter (%)	
	Shoots	Roots	Shoots	Roots	Shoots	Roots
Control	18 ± 2 c	12 ± 1 b	470.4 ± 47.0 e	33.6 ± 3.4 g	7.6 ± 0.8 b	7.3 ± 0.7 c
Se	18 ± 2 c	12 ± 1 b	1283.2 ± 128.3 d	54.4 ± 5.4 f	8.8 ± 0.9 ab	10.9 ± 1.1 ab
I	20 ± 2 bc	13 ± 1 b	1622.4 ± 162.1 c	105.6 ± 10.6 e	8.3 ± 0.8 ab	7.4 ± 0.7 c
Se + I	21 ± 2 abc	17 ± 2 a	1672.0 ± 167.0 c	288.0 ± 28.8 c	9.6 ± 0.9 a	10.0 ± 1.0 ab
Si	20 ± 2 bc	17 ± 2 a	2278.4 ± 228.0 b	192.0 ± 19.2 d	7.8 ± 0.8 b	8.5 ± 0.9 bc
Si + Se	22 ± 2 ab	19 ± 2 a	2622.4 ± 262.0 ab	448.0 ± 44.8 a	8.9 ± 0.9 ab	9.5 ± 0.9 b
Si + I	25 ± 3 a	20 ± 2 a	2928.0 ± 292.8 a	404.8 ± 40.4 ab	8.6 ± 0.8 ab	9.3 ± 0.9 b
Si + I + Se	22 ± 2 ab	21 ± 2 a	1851.2 ± 185.0 c	336.0 ± 33.6 bc	10.3 ± 1.0 a	11.7 ± 1.2 a

Within each column, values with the same letters do not differ according to Duncan test at *p* < 0.05.

**Table 2:** Photosynthetic pigments in chervil plants under Se, I and Si supplementation.

Treatment	Chlorophyll a (mg g^−1^ Fresh Weight)	Chlorophyll b (mg g^−1^ Fresh Weight)	Total Chlorophyll (mg g^−1^ Fresh Weight)	Chl b/a Ratio	Carotene (mg g^−1^ Fresh Weight)	Carotene:Chlorophyll Ratio
Control	0.70 ± 0.07 b	1.77 ± 0.18 b	2.47 ± 0.24 b	2.53 a	0.38 ± 0.04 b	1:6.66
Se	0.72 ± 0.07 b	1.98 ± 0.19 ab	2.70 ± 0.27 b	2.75 a	0.43 ± 0.04 ab	1:6.58
I	0.71 ± 0.07 b	1.87 ± 0.19 b	2.58 ± 0.25 b	2.63 a	0.40 ± 0.04 ab	1:6.58
Se + I	0.90 ± 0.09 a	2.18 ± 0.21 ab	3.08 ± 0.31 ab	2.42 a	0.46 ± 0.04 a	1:5.26
Si	0.88 ± 0.08 a	2.23 ± 0.22 a	3.11 ± 0.31 a	2.53 a	0.48 ± 0.05 a	1:5.27
Si + Se	0.96 ± 0.09 a	2.34 ± 0.23 a	3.30 ± 0.33 a	2.44 a	0.48 ± 0.05 a	1:5.08
Si + I	0.83 ± 0.08 ab	2.39 ± 0.23 a	3.22 ± 0.32 a	2.88 a	0.43 ± 0.04 ab	1:6.70
Si + Se + I	0.83 ± 0.08 ab	2.29 ± 0.22 a	3.12 ± 0.31 a	2.76 a	0.49 ± 0.05 a	1:5.63

Within each column, values with the same letters do not differ according to Duncan test at *p* < 0.05.

**Table 3:** Total antioxidant activity and phenolic content of chervil leaves under Se, I and Si supplementation.

Treatment	Ascorbic Acid (mg 100 g^−1^ Fresh Weight)	Antioxidant Activity-AOA (mg GAE g^−1^ Dry Weight)	Total Polyphenols-TP (mg GAE g^−1^ Dry Weight)
Control	30.0 ± 3.1 b	20.2 ± 2.0 bc	7.2 ± 0.71 c
Se	34.2 ± 3.4 b	24.6 ± 2.4 b	9.8 ± 0.9 ab
I	29.8 ± 3.0 b	24.0 ± 2.3 b	9.5 ± 0.9 ab
Se + I	30.4 ± 3.0 b	22.3 ± 2.2 b	10.3 ± 1.0 a
Si	31.4 ± 3.1 b	23.7 ± 2.3 b	8.4± 0.8 bc
Si + Se	28.5 ± 2.8 b	23.7 ± 2.3 b	9.4 ± 0.9 ab
Si + I	39.0 ± 3.8 a	19.1 ± 1.9 cd	8.9 ± 0.9 ab
Si + Se + I	25.7 ± 2.6 c	30.0 ± 3.0 a	11.3 ± 1.0 a

Within each column, the values with the same letters do not differ according to Duncan test at *p* < 0.05.

**Table 4:** Levels of Se, I and Si accumulation in chervil shoots under single and joint application of these elements.

Treatment	Se	I	Si
Control	0.23 ± 0.02 f	0.20 ± 0.02 e	20.59 ± 2.05 ab
Se	3.75 ± 0.32 ab	2.50 ± 0.21 c	23.81 ± 2.34 a
I	0.80 ± 0.07 c	16.00 ± 1.59 b	21.97 ± 2.20 a
Se + I	4.36 ± 0.41 a	15.00 ± 1.51 b	17.22 ± 1.71 b
Si	0.45 ± 0.04 e	1.50 ± 0.15 d	24.10 ± 2.40 a
Si + Se	3.73 ± 0.35 ab	1.60 ± 0.15 d	20.14 ± 2.00 ab
Si + I	0.62 ± 0.06 d	34.00 ± 3.38 a	19.66 ± 2.00 ab
Si + Se + I	3.07 ± 030 b	35.00 ± 3.51 a	17.16 ± 1.70 b

Se, I, Si are expressed in mg kg^−1^ dry weight. Within each column, values with the same letters do not differ according to Duncan test at *p* < 0.05.

**Table 5:** Levels of macro-element accumulation in chervil shoots under single and joint application of Se, I and Si.

Treatment	Ca	K	Mg	Na	P
Control	22.45 ± 2.22 bc	97.24 ± 9.71 a	4.35 ± 0.43 a	1.17 ± 0.11 a	7.86 ± 0.78 a
Se	20.09 ± 20.00 c	108.78 ± 10.80 a	3.76 ± 0.37 a	0.94 ± 0.09 a	8.36 ± 0.83 a
I	19.03 ± 19.0 c	91.07 ± 9.10 ab	3.51 ± 0.35 a	0.53 ± 0.05 d	6.70 ± 0.67 a
Se + I	25.22 ± 2.52 ab	88.22 ± 8.82 b	4.01 ± 0.40 a	0.76 ± 0.08 b	6.24 ± 0.62 a
Si	22.35 ± 2.23 bc	83.17 ± 8.31 ba	4.11 ± 0.40 a	0.72 ± 0.07 bc	6.95 ± 0.69 a
Si + Se	22.15 ± 2.21 bc	88.16 ± 8,80 ba	3.66 ± 0.36 a	0.44 ± 0.04 d	6.37 ± 0.63 a
Si + I	27.42 ± 2.74 a	100.78 ± 10.11 a	4.31 ± 0.43 a	0.71 ± 0.07 bc	7.37 ± 0.74 a
Si + Se + I	25.01 ± 2.50 ab	96.80 ± 9.66 a	3.84 ± 0.38 a	0.62 ± 0.06 c	7.21 ± −0.71 a

Ca, K, Mg, Na and P are expressed in g kg^−1^ dry weight. Within each column, values with the same letters do not differ according to Duncan test at *p* < 0.05.

**Table 6:** Levels of micro-element accumulation in chervil shoots under single and joint application of Se, I and Si.

Treatment	B	Co	Cu	Fe	Li	Mn	Mo	Zn
Control	32.18 ± 3.21 ab	0.42 ± 0.04 bc	21.33 ± 2.11 a	1323 ± 132 b	0.93 ± 0.09 ab	61.82 ± 6.18 b	1.85 ± 0.18 a	244 ± 24 a
Se	33.77 ± 3.37 ab	0.33 ± 0.03 d	18.85 ± 1.88 ab	776 ± 77 d	0.76 ± 0.07 bc	43.61 ± 4.36 c	1.43 ± 0.14 b	143 ± 14 bc
I	27.55 ± 2.75 b	0.33 ± 0.03 d	15.57 ± 1.55 c	753 ± 75 d	0.65 ± 0.06 c	42.94 ± 4.29 c	1.46 ± 0.15 b	121 ± 12 c
Se + I	33.29 ± 3.33 ab	0.52 ± 0.05 b	16.78 ± 1.67 c	1486 ± 148 b	1.06 ± 0.11 a	66.54 ± 6.65 b	2.06 ± 0.20 a	202 ± 20 a
Si	31.34 ± 3.13 ab	0.77 ± 0.07 a	16.76 ± 1.67 c	1880 ± 188 a	1.01 ± 0.10 a	93.71 ± 9.37 a	1.69 ± 0.17 a	166 ± 16 b
Si + Se	35.25 ± 3.52 a	0.39 ± 0.04 cd	14.16 ± 1.41 c	1237 ± 124 bc	0.87 ± 0.09 b	46.10 ± 4.60 c	1.80 ± 0.18 a	118 ± 12 c
Si + I	34.07 ± 3.40 a	0.49 ± 0.05 b	17.47 ± 1.75 a	1161 ± 116 c	0.92 ± 0.09 ab	62.30 ± 6.22 b	1.90 ± 0.19 a	145 ± 15 bc
Si + Se + I	36.42 ± 3.62 a	0.51 ± 0.05 b	17.14 ± 1.71 bc	1306 ± 130 bc	1.09 ± 0.11 a	63.81 ± 6.38 b	1.95 ± 0.19 a	161 ± 16 b

B, Co, Cu, Fe, Li, Mn, Mo and Zn are expressed in mg kg^−1^ dry weight. Within each column, values with the same letters do not differ according to Duncan test at *p* < 0.05.

**Table 7:** Levels of As, Al, Cd, Cr, Ni, Pb, Sr and V accumulation in chervil shoots under single and joint application of Se, I and Si.

Treatment	Al	As	Cd	Cr	Ni	Pb	Sr	V
Control	364 ± 36 de	0.28 ± 0.03 d	1.09 ± 0.11 b	13.39 ± 1.3 bc	3.51 ± 0.35 ab	3.59 ± 0.36 a	105.0 ± 10.0 a	1.33 ± 0.13 cd
Se	350 ± 35 e	0.23 ± 0.02 e	1.15 ± 0.11 ab	9.14 ± 0.91 d	3.33 ± 0.33 bc	2.55 ± 0.25 b	81.5 ± 8.1 b	1.04 ± 0.10 e
I	305 ± 30 e	0.27 ± 0.03 de	1.06 ± 0.10 b	8.29 ± 0.82 d	3.02 ± 0.30 c	2.67 ± 0.26 b	87.5 ± 8.7 ab	1.12 ± 0.11 de
Se + I	568 ± ab	0.41 ± 0.04 b	1.04 ± 0.10 b	14.75 ± 1.43 ab	3.68 ± 0.36 ab	3.53 ± 0.25 a	112.0 ± 11.1 a	1.79 ± 0.18 b
Si	513 ± 51 bc	0.71 ± 0.07 a	1.17 ± 0.11 ab	17.70 ± 1.76 a	3.43 ± 0.34 ab	3.55 ± 0.25 a	109.0 ± 10.7 a	2.32 ± 0.23 a
Si + Se	504 ± 50 bc	0.32 ± 0.03 cd	1.03 ± 0.10 b	12.81 ± 1.26 c	3.20 ± 0.32 cb	2.41 ± 0.24 b	82.8 ± 8.2 b	1.52 ± 0.15 c
Si + I	431 ± 43 cd	0.32 ± 0.03 cd	1.30 ± 0.13 a	12.91 ± 1.29 c	4.26 ± 0.42 a	2.93 ± 0.29 b	94.1 ± 9.4 ab	1.49 ± 0.15 c
Si + Se + I	660 ± 66 a	0.34 ± 0.03 bc	1.35 ± 0.13 a	14.01 ± 1.40 bc	3.93 ± 0.39 a	3.78 ± 0.38 a	99.4 ± 9.9 ab	1.95 ± 0.19 ab

Al, As, Cd, Cr, Ni, Pb, Sr, V are expressed in mg kg^−1^ dry weight. Within each column, values with the same letters do not differ according to Duncan test at *p* < 0.05.

## Data Availability

Not applicable.

## Supplementary Materials

The following are available online at https://www.mdpi.com/article/10.3390/plants10112528/s1, Table S1: Coefficients of correlation between mineral elements in chervil shoots.
