# Supplementary material for: Iodine and Selenium Biofortification of Chervil Plants Treated with Silicon Nanoparticles
Source: Plants (Basel). 2021 Nov 20;10(11):2528. doi: 10.3390/plants10112528 (PMC8618568; doi:10.3390/plants10112528)
Supplement: Supplementary file 1 [file plants-10-02528-s001.zip › plants-1473353-supdone.pdf]

Table S1. Coefficients of correlation between mineral elements in chervil shoots.

	**Al**	**As**	**B**	**Ca**	**Cd**	**Co**	**Cr**	**Cu**	**Fe**	**I**	**K**	**Li**	**Mg**	**Mn**	**Na**	**Ni**	**P**	**Pb**	**Se**	**Si**	**Sr**	**V**
As	0.432	1																				
B	0.666	-0.108	1																			
Ca	0.606	0.163	0.590	1																		
Cd	0.384	0.070	0.459	0.554	1																	
Co	0.555	0.953a	0.070	0.412	0.303	1																
Cr	0.688d	0.822b	0.315	0.552	0.199	0.909a	1															
Cu	-0.302	-0.204	0.015	0.059	0.185	-0.054	0.015	1														
Fe	0.626	0.866c	0.197	0.442	0.082	0.914a	0.989a	-0.006	1													
I	0.391	-0.171	0.282	0.653	0.733d	0.038	-0.021	-0.147	-0.126	1												
K	-0.449	-0.542	0.164	-0.180	0.479	-0.457	-0.526	0.424	-0.592	0.113	1											
**Li**	0.869c	0.513	0.596	0.742d	0.394	0.698d	0.867c	0.118	0.807b	0.283	-0.426	1										
Mg	0.079	0.285	0.175	0.629	0.307	0.481	0.583	0.649	0.538	0.073	0.006	0.538	1									
Mn	0.484	0.907a	0.028	0.420	0.293	0.979a	0.911a	0.133	0.918a	0.008	-0.423	0.716d	0.610	1								
Na	-0.340	-0.125	-0.038	-0.017	-0.014	-0.025	0.054	0.968a	0.057	-0.340	0.288	0.096	0.638	0.158	1							
Ni	0.463	0.052	0.557	0.936a	0.761e	0.336	0.398	0.260	0.273	0.738d	0.114	0.633	0.657	0.368	0.135	1						
P	-0.438	-0.350	0.111	-0.158	0.383	-0.247	-0.303	0.788e	-0.347	-0.106	0.843b	-0.214	0.302	-0.141	0.720d	0.148	1					
Pb	0.484	0.470	0.144	0.420	0.357	0.640	0.703d	0.421	0.687d	0.237	-0.340	0.808b	0.566	0.737d	0.364	0.464	-0.022	1				
Se	0.177	0.486	-0.271	-0.202	-0.626	0.265	0.244	-0.772e	0.333	-0.351	-0.773	-0.031	-0.394	0.143	-0.608	-0.493	-0.844b	-0.231	1			
Si	-0.673	0.001	-0.435	-0.521	-0.282	-0.071	-0.092	0.635	-0.023	-0.689d	0.366	-0.358	0.312	0.055	0.685d	-0.402	0.580	-0.026	-0.264	1		
Sr	0.461	0.625	-0.040	0.470	0.070	0.731d	0.785b	0.305	0.801b	0.067	-0.618	0.758e	0.639	0.820b	0.338	0.375	-0.237	0.898a	0.074	-0.006	1	
**V**	0.795b	0.875c	0.263	0.465	0.316	0.934a	0.922a	-0.224	0.913a	0.145	-0.543	0.816b	0.306	0.883c	-0.218	0.336	-0.418	0.672	0.329	-0.304	0.693	1
Zn	0.096	0.122	0.032	0.266	-0.086	0.251	0.444	0.762e	0.454	-0.190	-0.224	0.516	0.714d	0.428	0.794e	0.254	0.220	0.740d	-0.323	0.393	0.759e	0.186
**Mo**	0.722d	0.176	0.557	0.857b	0.214	0.375	0.650	0.036	0.580	0.412	-0.475	0.848b	0.530	0.407	0.006	0.680d	-0.387	0.612	0.002	-0.468	0.634	0.527

a) P<0.001; b) P<0.01; c) P<0.002; d) P<0.05; e) P<0.02
